# Supplementary material for: Spatiotemporal Heterogeneity in the Distribution of Chikungunya and Zika Virus Case Incidences during their 2014 to 2016 Epidemics in Barranquilla, Colombia
Source: Int J Environ Res Public Health. 2019 May 18;16(10):1759. doi: 10.3390/ijerph16101759 (PMC6572372; doi:10.3390/ijerph16101759)
Supplement: Supplementary file 1 [file ijerph-16-01759-s001.pdf]

Supplementary file

Table S1. Neighbourhood names with their corresponding codes, as used to the Barranquilla map (Figure 1).

Code	Neighborhood	Code	Neighborhood	Code	Neighborhood	Code	Neighborhood
**0802**	ABAJO	**0311**	EL POBLADO	**2602**	LAS MALVINAS	**2802**	SAN LUIS
**1803**	ALFONSO LOPEZ	**0504**	EL PORVENIR	**1105**	LAS MERCEDES	**3601**	SAN NICOLAS
**0310**	ALTAMIRA	**0602**	EL PRADO	**1103**	LAS MERCEDES SUR	**1901**	SAN ROQUE
**0503**	ALTOS DEL PRADO	**1206**	EL PUEBLO	**2201**	LAS NIEVES	**0104**	SAN SALVADOR
**0301**	ALTOS DEL RIOMAR	**1001**	EL RECREO	**3503**	LAS PALMAS	**0307**	SAN VICENTE
**0902**	AMERICA	**3203**	EL SANTUARIO	**1101**	LAS TERRAZAS	**0704**	SANTA ANA
**0302**	ANDALUCIA	**1401**	EL SILENCIO	**0203**	LAS TRES AVEMARIAS	**3701**	SANTA ELENA
**1802**	ATLANTICO	**0313**	EL TABOR	**2604**	LIPAYA	**2901**	SANTA MARIA
**0803**	BARLOVENTO	**2504**	EL VALLE	**1702**	LOMA FRESCA	**0306**	SANTA MONICA
**0013**	BELLA ARENA	**0308**	GRANADILLO	**0402**	LOS ALPES	**1405**	SANTO DOMINGO
**0601**	BELLAVISTA	**3602**	JOSE ANTONIO GALAN	**1601**	LOS ANDES	**2803**	SANTO DOMINGO DE GUZMAN
**1106**	BETANIA	**3202**	KENNEDY	**3704**	LOS ANGELES	**0103**	SIAPE
**0903**	BOSTON	**3302**	LA ALBORAYA	**2405**	LOS CONTINENTES	**2902**	SIETE DE ABRIL
**3401**	BOYACA	**0309**	LA CAMPINA	**1104**	LOS HOBOS	**3702**	SIMON BOLIVAR
**1605**	BUENA ESPERANZA	**2403**	LA CEIBA	**0020**	LOS LAURELES	**0303**	SOLAIRE
**3501**	BUENOS AIRES	**2105**	LA CHINITA	**0405**	LOS NOGALES	**2603**	SOURDIS
**0406**	CAMPO ALEGRE	**0702**	LA CONCEPCION	**1204**	LOS OLIVOS	**3504**	TAYRONA
**2501**	CARLOS MEISEL	**2401**	LA CUCHILLA DE VILLATE	**1602**	LOS PINOS	**3604**	UNIVERSAL
**3001**	CARRIZAL	**0401**	LA CUMBRE	**1701**	LUCERO	**3606**	VILLA BLANCA
**0804**	CENTRO	**2503**	LA ESMERALDA	**1302**	ME QUEJO	**0502**	VILLA COUNTRY
**2303**	CEVILLAR	**0202**	LA FLORESTA	**0705**	MODELO	**0304**	VILLA DEL ESTE
**1801**	CHIQUINQUIRA	**1102**	LA FLORIDA	**0801**	MONTECRISTO	**1203**	VILLA ROSARIO
**0404**	CIUDAD JARDIN	**1402**	LA LIBERTAD	**1902**	MONTES	**2801**	VILLA SAN PEDRO
**2601**	CIUDAD MODESTO	**2101**	LA LOMA	**2502**	NUEVA COLOMBIA	**0312**	VILLA SANTOS
**1603**	CIUDADEA DE LA SALUD	**2104**	LA LUZ	**1404**	NUEVA GRANADA	**2102**	VILLANUEVA
**3101**	CIUDADELA 20 DE JULIO	**3502**	LA MAGDALENA	**0403**	NUEVO HORIZONTE	**2402**	VILLATE
**0901**	COLOMBIA	**1303**	LA MANGA	**1403**	OLAYA	**2103**	ZONA INDUSTRIAL I
**2701**	EL BOSQUE	**1205**	LA PAZ	**3703**	PASADENA	**0102**	ZONA INDUSTRIAL II
**3403**	EL CAMPITO	**1201**	LA PRADERA	**1301**	POR FIN	**0701**	ZONA FRANCA
**2301**	EL CARMEN	**2404**	LA SIERRA	**1604**	PUMAREJO		
**0205**	EL CASTILLO I	**3201**	LA SIERRITA	**2001**	REBOLO		
**0204**	EL CASTILLO II	**3402**	LA UNION	**0305**	RIOMAR		
**0007**	EL FERRY	**3301**	LA VICTORIA	**0805**	ROSARIO		
**0501**	EL GOLF	**2804**	LAS AMERICAS	**1501**	SAN FELIPE		
**3603**	EL LIMON	**1107**	LAS DELICIAS	**0703**	SAN FRANCISCO		
**0201**	EL LIMONCITO	**1202**	LAS ESTRELLAS	**1703**	SAN ISIDRO		
**0206**	EL PARAISO	**0101**	LAS FLORES	**2302**	SAN JOSE		

Figure S1. Satellite image from Landsat 8 mission for the Barranquilla area obtained in December 2014. The image shows maps of natural band combination (4-3-2) and infrared colour combination (5-4-3), and the derived products that resulted from the supervised classification and calculation of the MNDWI.

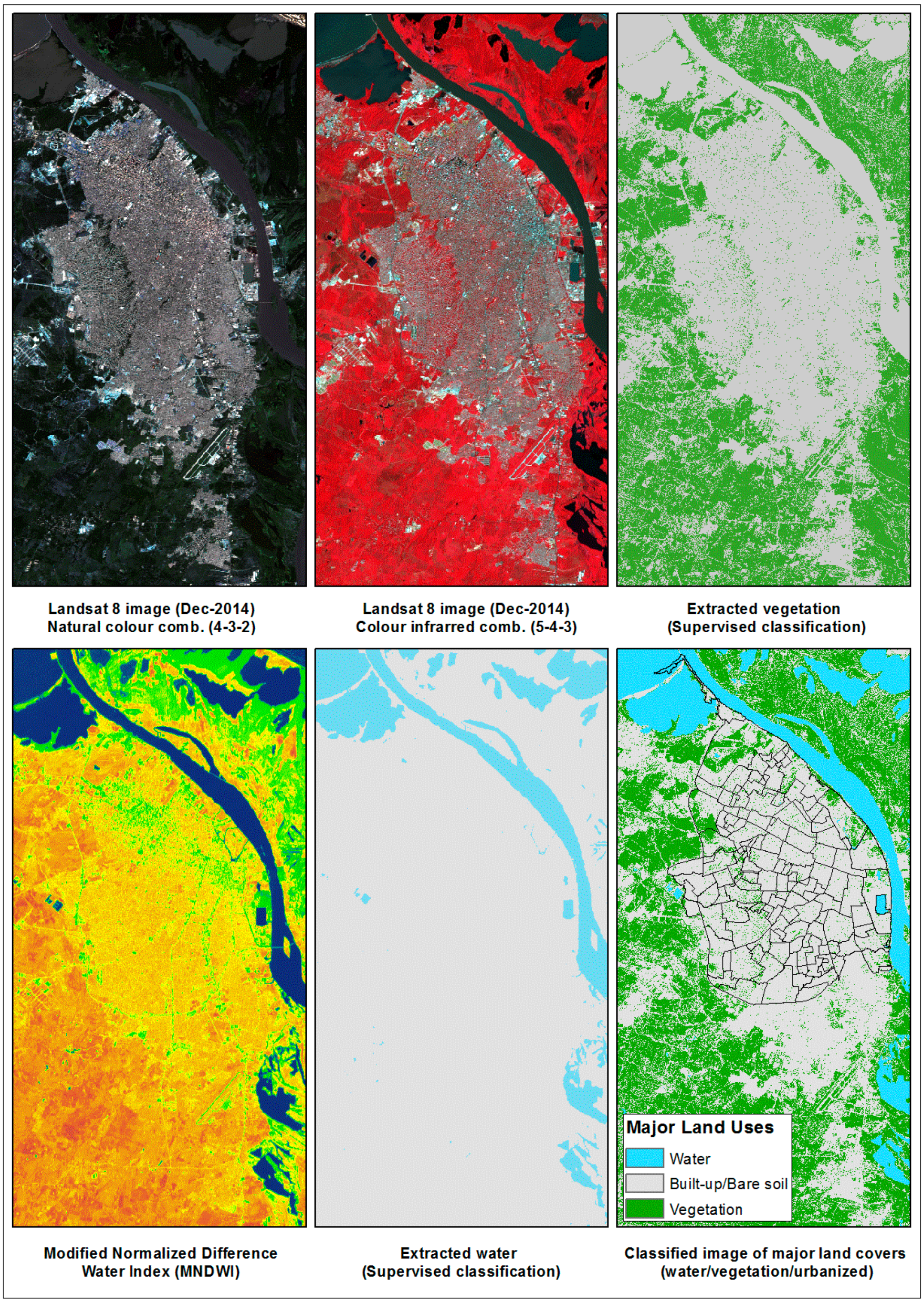

Figure S2. Satellite image from Landsat 8 mission for the Barranquilla area obtained in December 2015. The image shows maps of natural band combination (4-3-2) and infrared colour combination (5-4-3), and the derived products that resulted from the supervised classification and calculation of MNDWI.

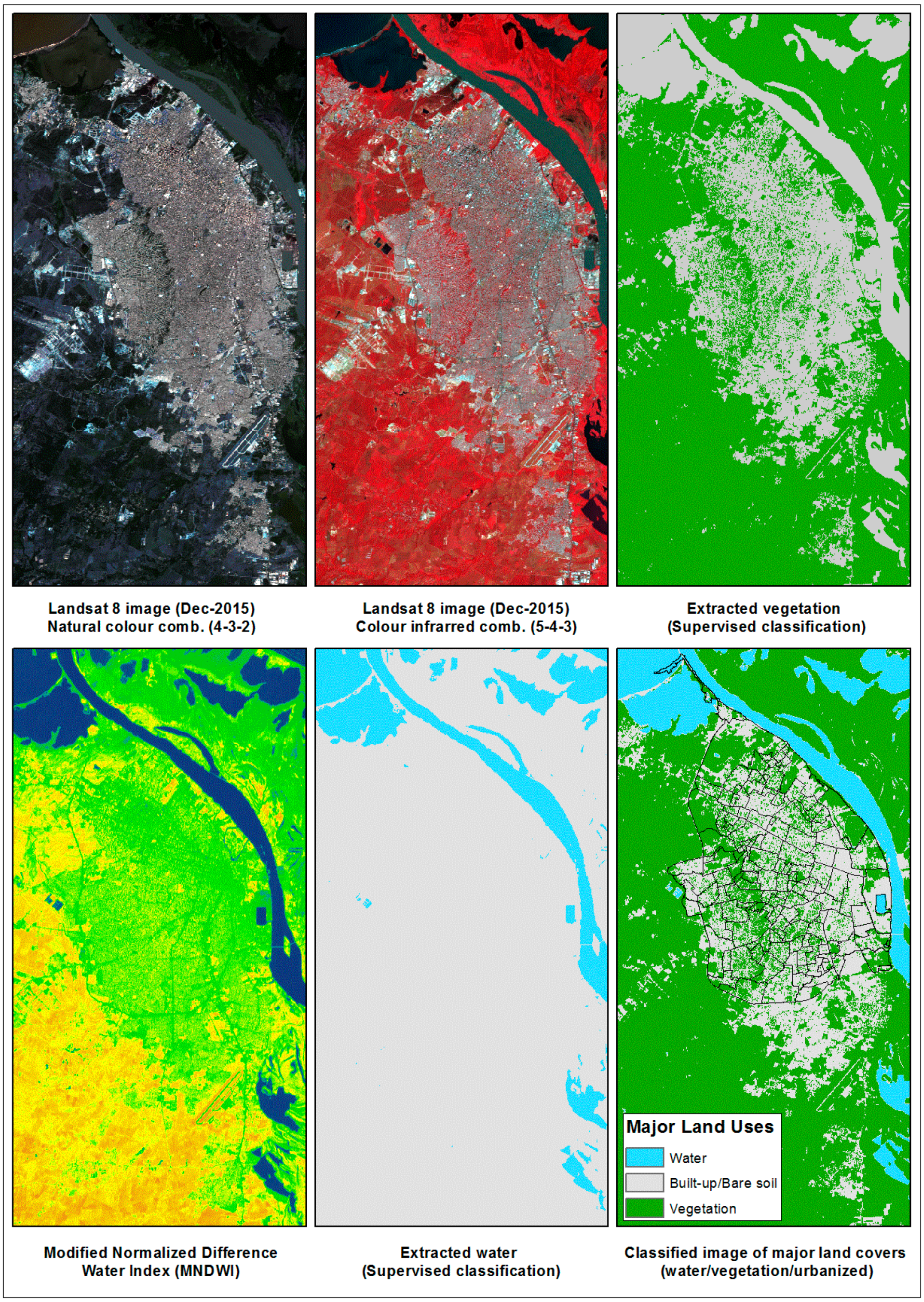

Figure S3. Incidence of CHIKV infected case notifications to SIVIGILA (N.B. the case definitions are described in the Methods section). Each bar represents one week.

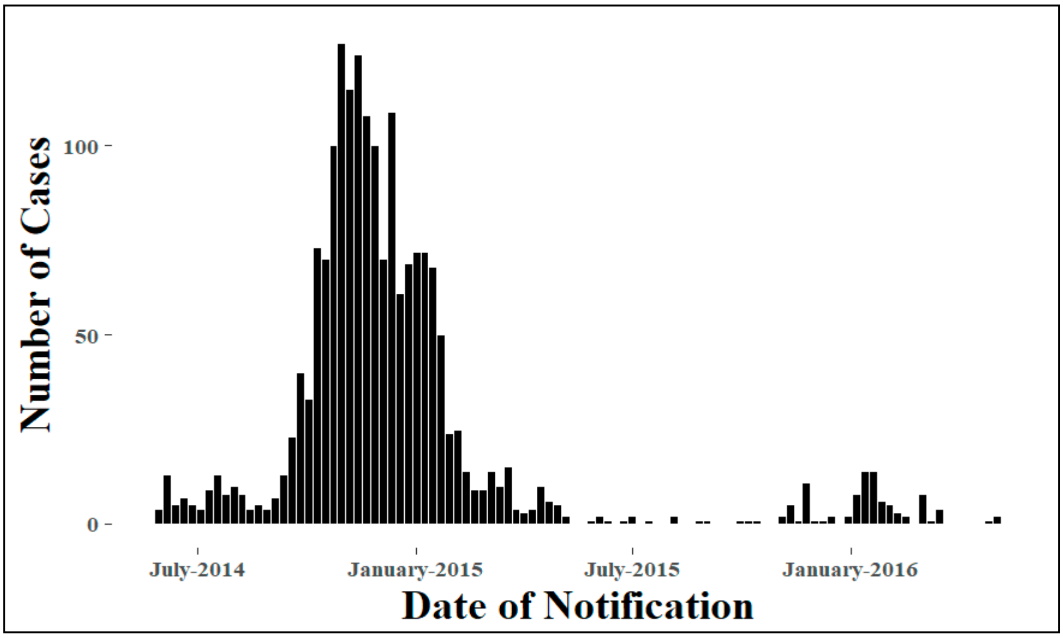

Figure S4. The estimated incidence of CHIKV infected cases per 10,000 residents by neighbourhood in Barranquilla between 2014 and 2015.

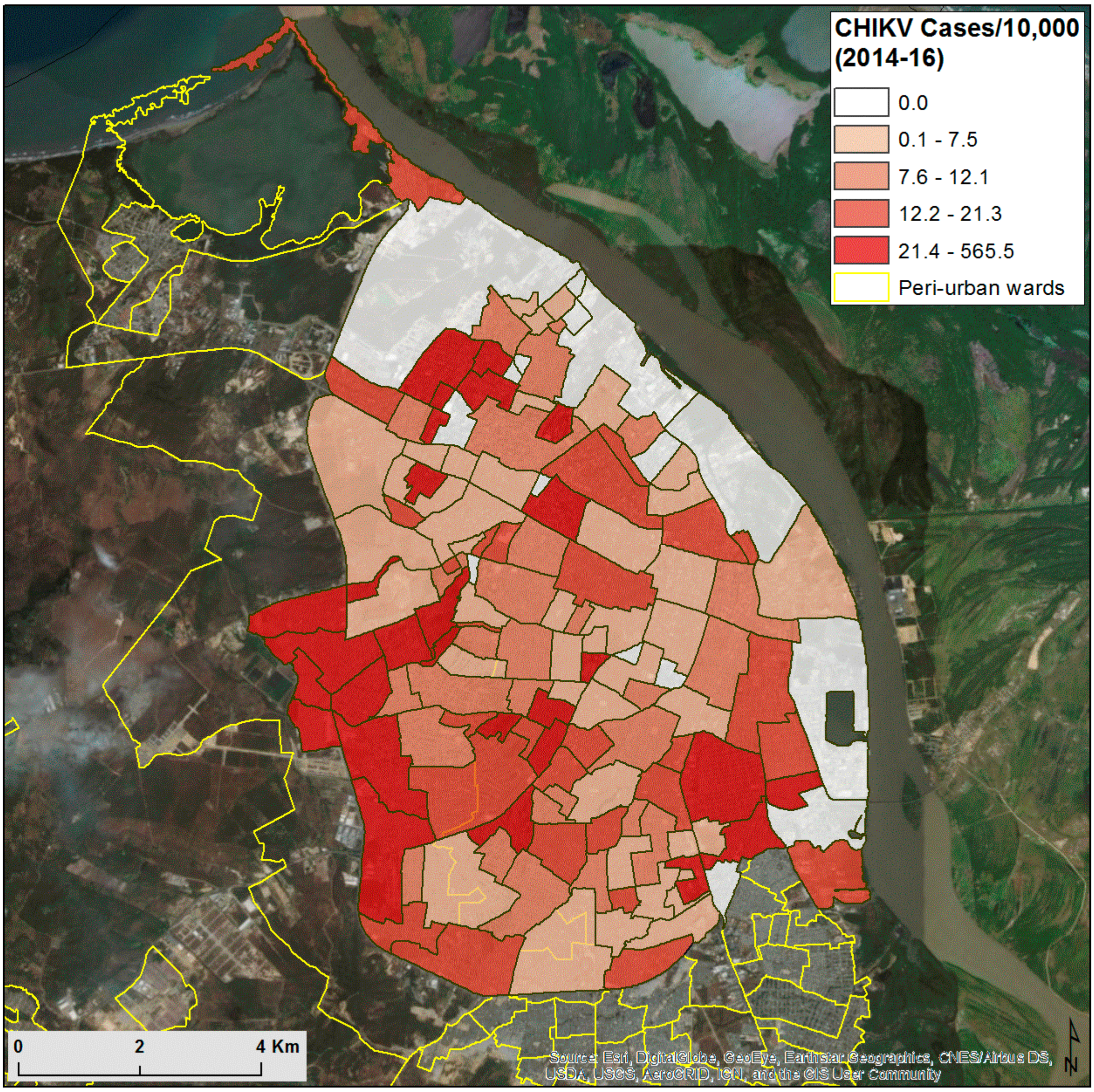

Figure S5. The univariate Local Moran's I (LISA) cluster map of the overall incidence of CHIKV infected cases during 2014-2016.

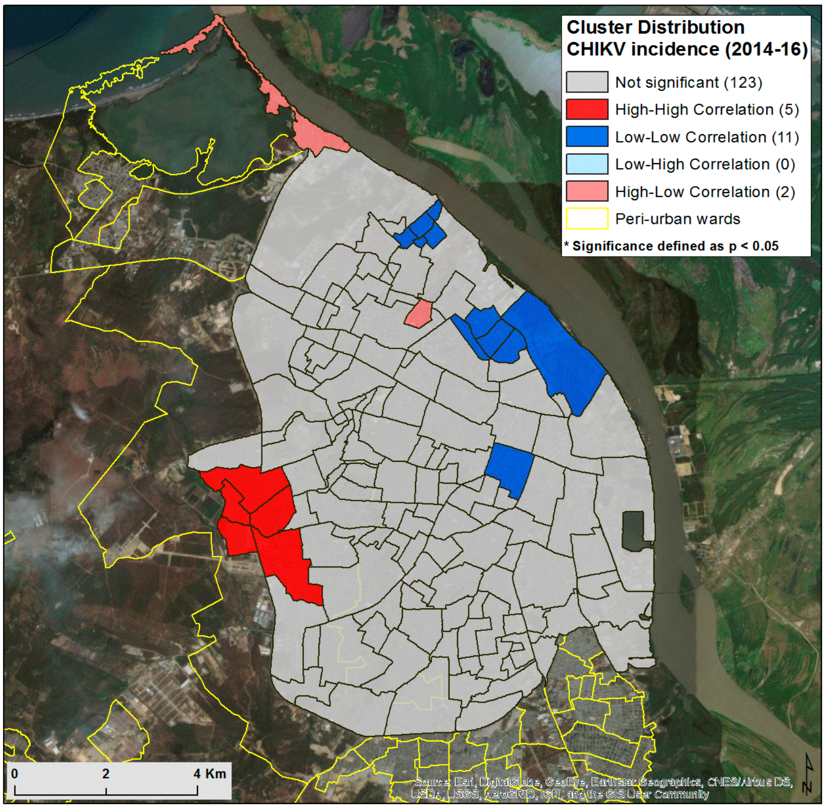

Figure S6. Incidence of ZIKV infected case notifications to SIVIGILA (N.B. the case definitions described in methods). Each bar represents one week.

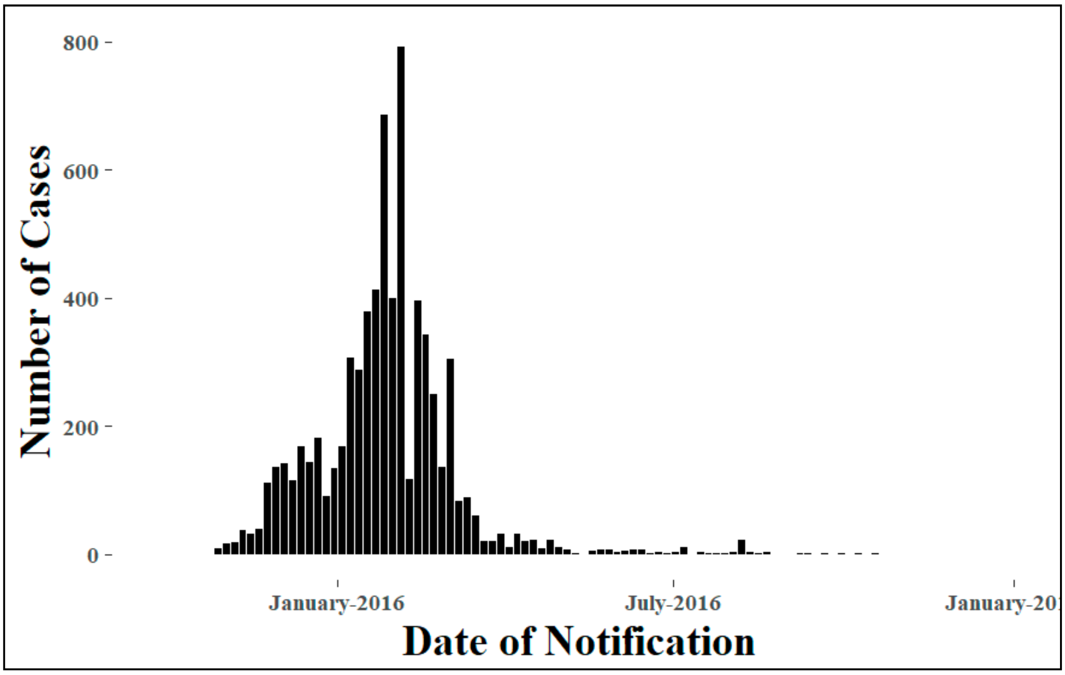

Figure S7. The estimated incidence of ZIKV infected cases per 10,000 residents by neighbourhood in Barranquilla between 2015 and 2016.

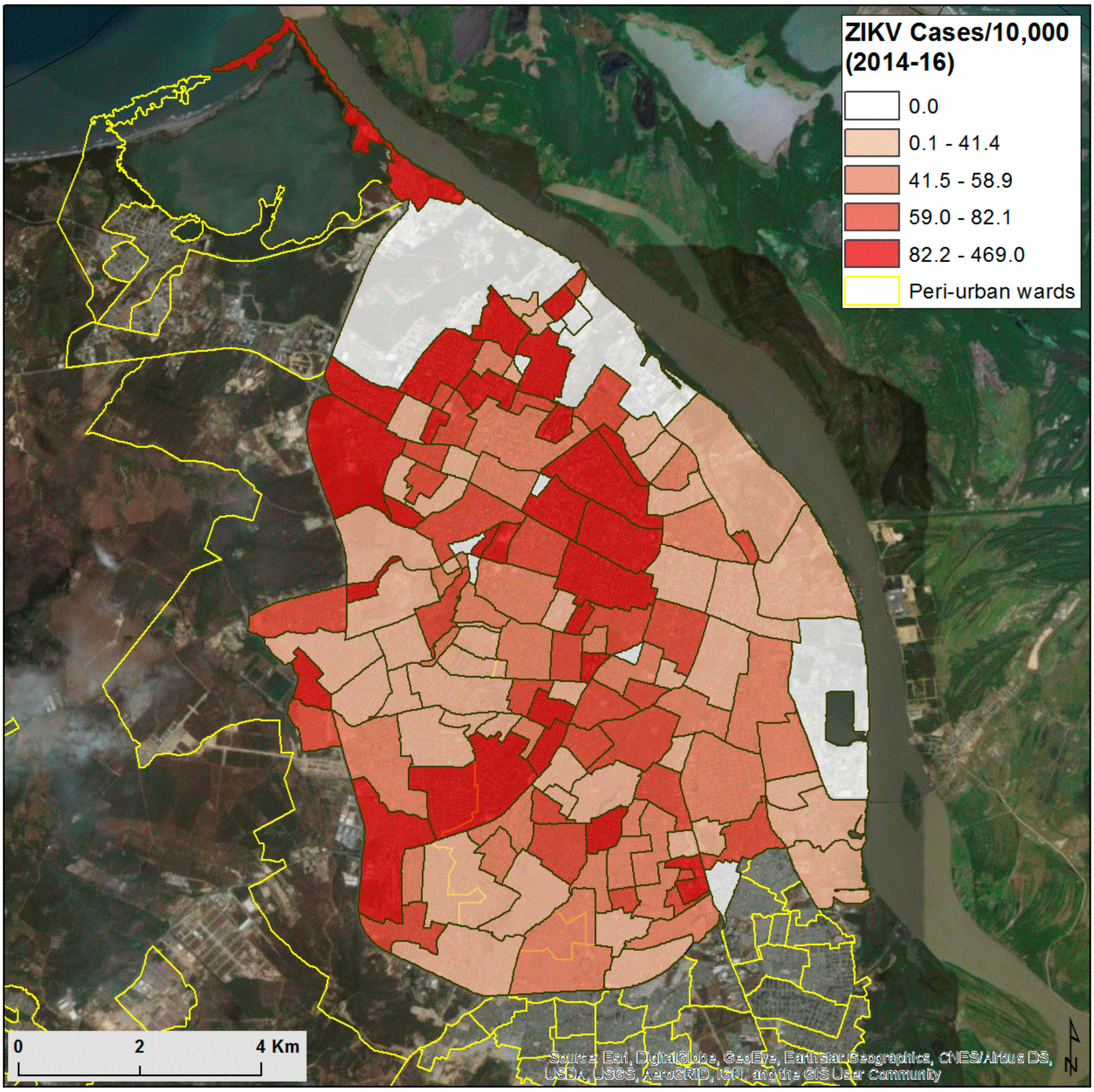

Figure S8. The univariate Local Moran's I (LISA) cluster map of the overall incidence of ZIKV infected cases during 2014-2016.

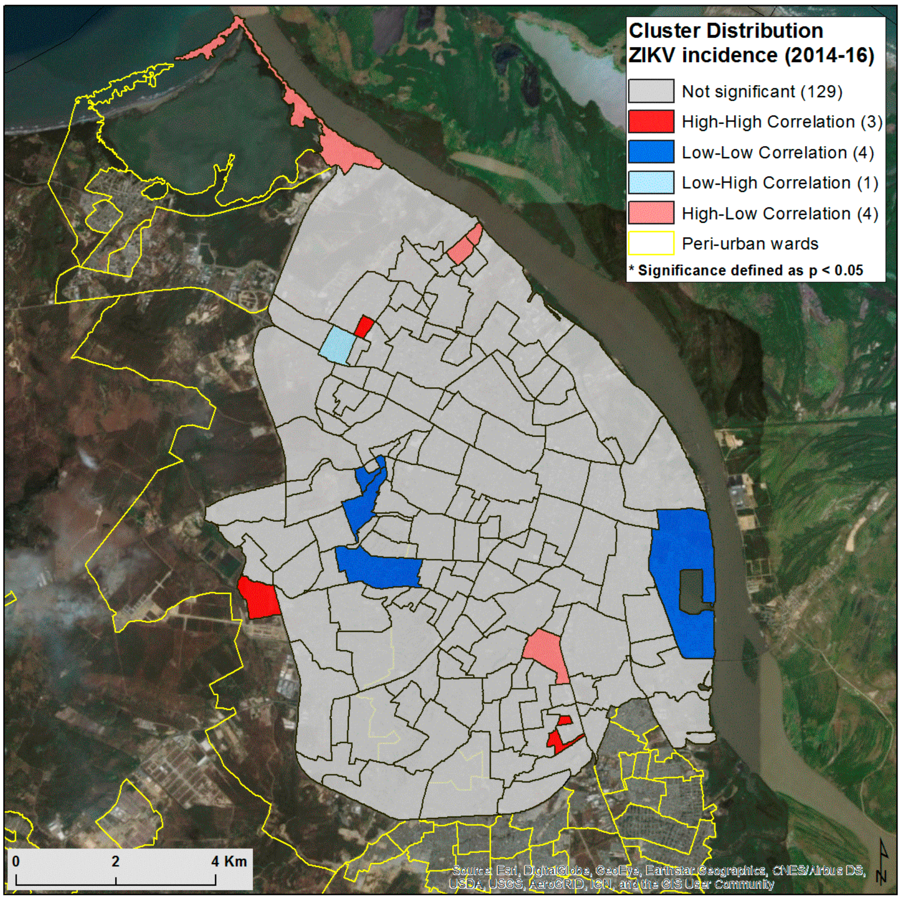

Figure S9. Local Moran's I analysis on the residuals resulting from fitting the ZIKV SIR values with spatial explanatory variables and independent random effects. Maps show (A) the persisting local clusters and (B) their corresponding p-values.

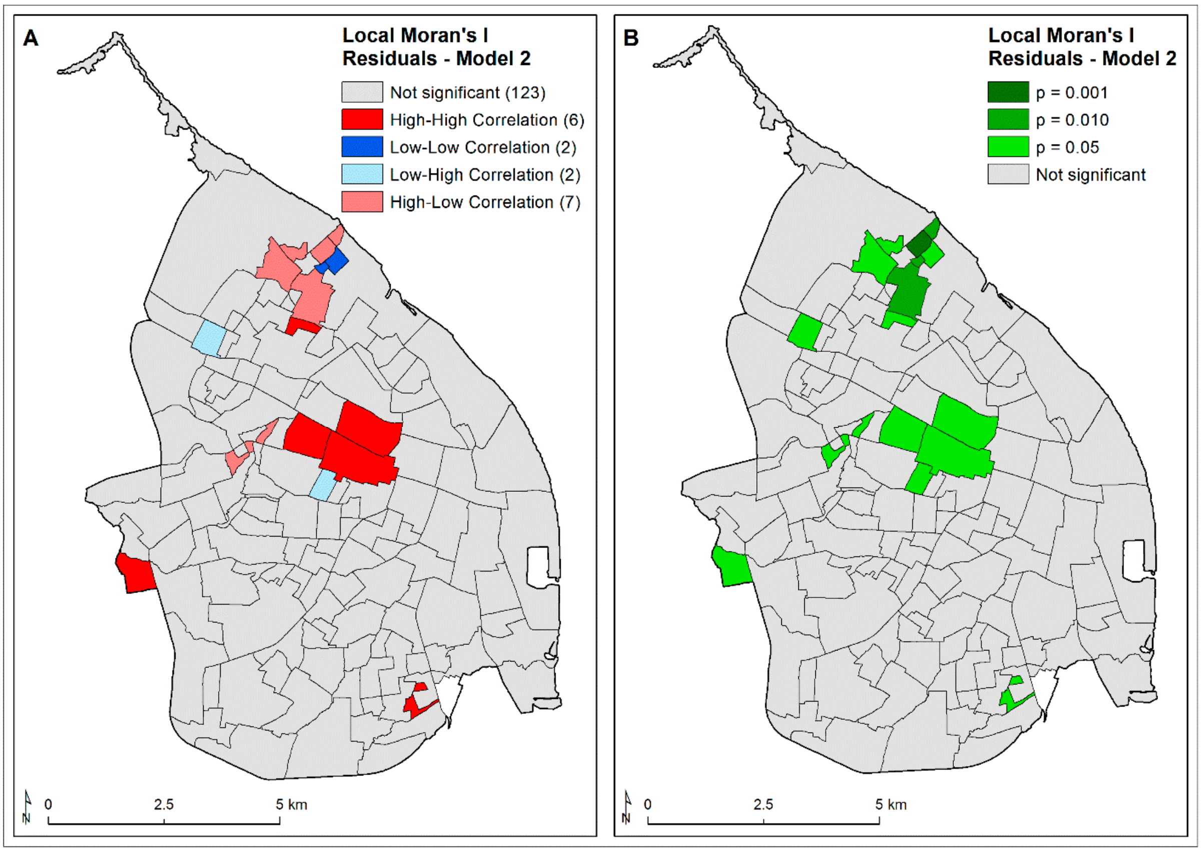

Text S1. Implementation of spatial autocorrelation analysis (Global Moran's I & Local Indicator of Spatial Autocorrelation, LISA)

Global Moran's I statistic provides an overall measure of spatial autocorrelation across the entire map of Barranquilla. This was calculated by first determining the relationship of each neighbourhood to its neighbours. In our analyses, we explored a sensitivity analysis of global spatial autocorrelation with 1st, 2nd, 3rd, and 4th order queen's contiguity. Queen's contiguity indicates that common sides and vertices of polygons or areas were considered to define the neighbour relation. The order indicates how many degrees of separation were allowed. For example, a 2nd order Queen's contiguity would include all adjacent neighbours and all the immediate neighbours of the original neighbours and so on. We determined that spatial autocorrelation disappeared after the 1st order and therefore we used 1st order Queen's contiguity throughout the analyses.

For the Moran's I statistic, the sum of covariations between the sites (neighbourhoods' centroids) for the distance d(i,j) is divided by the overall number of sites $W(d_{i,j})$ within the distance class d(i,j). Thus, the spatial autocorrelation coefficient for a distance class d(i,j) is the average value of spatial autocorrelation at that distance.

$$I = \frac{n}{S_p} \frac{\sum_{i=1}^{n}\sum_{j=1}^{n} W_{ij}(\gamma_i-\bar{\gamma})(\gamma_j-\bar{\gamma})}{\sum_{i=1}^{n}(\gamma_i-\bar{\gamma})^2}, \text{ where}$$

n = the sample size

$W_{ij} = \begin{cases} 1 \text{ if sites i, j are neighbours} \\ 0 \text{ otherwise} \end{cases}$ = row-standardized spatial weights matrix of sites i and j

$$S_p = \sum_{i=1}^{n}\sum_{j=1}^{n} W_{i.j} = \text{sum of the number of sampling locations per distance class,}$$

γ_i = the value at site i; $\bar{\gamma}$ = global mean value

The actual value for the Moran's I statistic was then compared with the expected value under the assumption of complete randomisation.

$$E(I) = -\frac{1}{n-1}$$

The lagged value of global Moran's I statistic was determined by calculating the mean of all 1st order neighbours. This value was then plotted against the incidence of each neighbourhood and a sum of least squares line was fitted. The slope of this line determined the Moran's I statistic. A Moran's I statistic value of 0 suggested complete spatial randomness. A value of 1 suggested very high spatial autocorrelation, while a value of -1 suggested spatial dispersion.

A local version, called local indicator of spatial association (LISA) or Anselin local Moran's I [1] allowed us to test for statistically significant local spatial clusters, including the type and location of these clusters. It is calculated as follows:

$$I_i(d) = \frac{(\gamma_i - \bar{\gamma})}{\frac{1}{n}\sum_{i=1}^{n}(\gamma_i - \bar{\gamma})} \sum_{i=1}^{n} W_{ij}(d)(\gamma_i - \bar{\gamma}), \text{ where}$$

$W_{ij}(d)$ was the row-standardized weights matrix given a local neighbourhood search radius *d.* The neighbourhood definition was the same as the one applied previously when we explored the overall spatial pattern for the ZIKV and CHIKV incidence across Barranquilla. Unlike the global Moran's I statistic, which had the same expected value for the entire study area, the expected value of local Moran's I statistic varied for each sampling area because it was calculated in relation to its particular set of neighbours.

$$E(I_i) = -\frac{1}{n-1}\sum_{j=1}^{n} W_{i,j}$$

Positive spatial autocorrelation occurred when, for example, an area with a specific outcome value was surrounded by neighbouring areas with similar outcome values (e.g. low-low, high-high), thus forming a spatial cluster.

The statistical evidence for both the global and local autocorrelation was evaluated using a pseudo p-value generated from a Monte Carlo randomisation method with 999 permutations. Neighbourhoods were displayed as significant in the LISA maps if they had p values of < 0.05 [2].

Text S2. Details on the implementation of Bayesian statistical modelling

The infected case incidence data were fitted using multiple Bayesian Poisson models based on a suite of explanatory variables as potential risk factors and different options for random effects with: i) no random effects, ii) independent random effects, and iii) spatially correlated random effects, implemented through a conditional autoregressive model (CAR).

Let us first introduce the general formulation

$$\begin{array}{ccc} Y_i|\mu_i & \sim & Poissson(\mu_i) i = 1, \dots, N \\ log(\mu_i) & = & x_i^T \beta + O_i + U_i \\ \beta & \sim & N(\mu_\beta, \Sigma_\beta) \end{array}$$

where x_i was a $(p+1) \times 1$ vector of known exploratory variables that were suspected to be risk factors for the disease, O_i were known offsets, in our case the expected number of cases, and U_i were random effects with a spatial structure to model the residual spatial variation that was not captured by the covariates.

1. The model without random effects. For this model, we initially fitted a Bayesian Poisson model with no random effects to the dataset. This corresponded to a model where $U_i = 0$ for each observation.

2. The model with independent random effects. To capture the extra Poisson variability present in the data, we introduced a set of independent and normally distributed random effects. This equated to define $U_i \sim N(0, \tau^2)$ in the general model previously introduced.

3. The globally smooth CAR model with spatially correlated random effects. For this model, we used the Besag-York-Mollie (BYM) model as described in Besag et al. (1991) [3]. In this case we had two sets of random effects (spatially structured and independent):

$$U_i = \phi_i + \theta_i$$

$$\theta_i \sim N(0, \sigma^2)$$

$$\phi_i \mid \phi_{-i} \sim N\left(\frac{\sum_{i=1}^{n} w_{ik}\phi_i}{\sum_{i=1}^{n} w_{ik}}, \frac{1}{\tau^2 \sum_{i=1}^{n} w_{ik}}\right)$$

$$\log \sigma, \log \tau \sim \log Gamma\ (1, 0.0005)$$

Weekly informative priors were specified on the log of the independent random effect precision value $1/\sigma$ and on the log of the spatially structured effect precision τ . Moreover, $w_{ik} = 0$ was obtained if the neighbourhood i and k did not share a border and visa-versa if $w_{ik} = 1$ was obtained. These values were fixed and calculated *a priori* based on the adjacency of neighbourhoods.

4. Locally smooth CAR model with spatially correlated random effects. The model that was implemented was described in Lee and Mitchell (2013) [4]. The random effects of the general model now had the following structure

$$\begin{aligned} U_i|U_{-i} &\sim N\left(\frac{\rho \sum_{i=1}^{n} w_{ik}\, U_i}{\rho \sum_{i=1}^{n} w_{ik} + 1 - \rho}, \frac{1}{\tau(\rho \sum_{i=1}^{n} w_{ik} + 1 - \rho)}\right) \\ logit(\rho) &\sim N(0{,}100) \end{aligned}$$

$$\tau \sim Gamma(0.001, 0.001)$$

where, ρ determined the global spatial correlation ($\rho = 0$ corresponded to independence everywhere, whereas $\rho = 1$ defined strong spatial correlation throughout the study region). Diffuse priors were assigned to both the precision parameter τ and the logit of ρ, Gamma and a Gaussian respectively. The neighborhood matrix elements w were usually fixed as for the BYM model previously specified. In this way it was not possible to capture localized spatial variation and discontinuities in the random effects surface. To solve this problem Lee and Mitchell (2013) [4] estimated the w_{ik} elements. This model was applied to the ZIKV case incidence because local autocorrelation was found in the residuals of the model with independent random effects.

REFERENCES

1. Anselin L. Local indicators of spatial association—LISA. Geogr Anal. 1995;27:93–115.
2. Ruxton GD, Neuhäuser M. Improving the reporting of P-values generated by randomization methods. Meths Ecol Evol. 2013;4:1033–6.
3. Besag J, York J, Mollie A. Bayesian image restoration, with two applications in spatial statistics. Ann Inst Stat Math. 1991;43:1–20.
4. Lee D, Mitchell R. Locally adaptive spatial smoothing using conditional auto-regressive models. J R Stat Soc Ser C (Appl Stat). 2013;62:593–608.
